# Theaflavin-3,3′-Digallate Inhibits Erastin-Induced Chondrocytes Ferroptosis via the Nrf2/GPX4 Signaling Pathway in Osteoarthritis

**DOI:** 10.1155/2022/3531995

**Published:** 2022-11-17

**Authors:** Chao Xu, Su Ni, Nanwei Xu, Guangrong Yin, Yunyuan Yu, Baojun Zhou, Gongyin Zhao, Liangliang Wang, Ruixia Zhu, Shijie Jiang, Yuji Wang

**Affiliations:** ^1^Truma Central, The Affiliated Changzhou No.2 People's Hospital with Nanjing Medical University, 29 Xinglong Alley, Changzhou 213003, China; ^2^Medical Research Center, The Affiliated Changzhou No.2 People's Hospital of Nanjing Medical University, 29 Xinglong Alley, Changzhou 213003, China; ^3^Department of Orthopedics, The Affiliated Changzhou Second People's Hospital of Nanjing Medical University, Changzhou Second People's Hospital, Changzhou Medical Center, Nanjing Medical University, 29 Xinglong Alley, Changzhou 213003, China; ^4^Graduate School of Dalian Medical University, 9 West Section, Shunnan Road, Dalian 116044, China; ^5^The Affiliated Changzhou No.2 People's Hospital of Nanjing Medical University, 29 Xinglong Alley, Changzhou 213003, China; ^6^Department of Orthopedics, The Third Affiliated Hospital of Gansu University of Chinese Medicine, 222 Silong Road, Baiyin 730900, China; ^7^Department of Orthopedic Surgery and Biochemistry & Molecular Biology, Mayo Clinic, Rochester, MN, USA

## Abstract

There is evidence that osteoarthritis (OA) is associated with ferroptosis which is a kind of lipid peroxidation-related cell death. Theaflavin-3,3′-digallate(TF3), a polyphenol compound extracted from black tea, possesses antioxidative and anti-inflammatory properties, but its effects on chondrocyte ferroptosis in osteoarthritis (OA) remain unclear. Our present study aims at exploring the protective role and underlying mechanisms of TF3 against erastin-induced chondrocyte ferroptosis in OA. In human primary chondrocytes treated with erastin alone or combined with different doses of TF3, cell viability was assessed by MTS. Ferroptosis-related proteins, including Gpx4, HO-1, and FTH1, were detected by western blot. The levels of lipid peroxidation and Fe2^+^ were determined by fluorescence staining. Meanwhile, the change of related proteins in the Nrf2/Gpx4 signaling pathway was determined by western blot. siRNA-mediated Nrf2 knockdown and the Gpx4 inhibitor RSL3 were used to explore molecular mechanisms for TF3-induced ferroptosis in OA chondrocyte. The magnetic resonance imaging (MRI), HE staining, Masson's staining, and immunohistochemistry were used to evaluate articular cartilage damages in the rat OA model. The results showed that Gpx4 expression was markedly downregulated in the chondrocytes of OA patients. TF3 reversed erastin-induced ferroptosis of human cultured chondrocytes, lipid ROS, and Fe2^+^ production in mitochondria. Moreover, the expression of Gpx4, HO-1, FTH1, and Nrf2 was markedly induced by TF3 in the erastin-treated chondrocytes. The antiferroptotic effect of TF3 was related to enhance Nrf2/Gpx4 signaling pathway. Finally, TF3 inhibited OA progression by alleviating *in vivo* cartilage damage related to chondrocyte ferroptosis. Thus, TF3 significantly inhibits chondrocyte ferroptosis by activating the Nrf2/Gpx4 signaling pathway, suggesting that TF3 serves as a potential therapeutic supplement for OA treatment.

## 1. Introduction

Osteoarthritis (OA), a chronic degenerative disease, has become a notable cause of disability in elderly patients. The incidence of OA among people over 75 years old is approximately 70% to 90%, and more than 100 million people worldwide suffer from OA [1]. The clinical manifestations of OA are joint pain, swelling, joint deformities, and restricted mobility [2]. Cartilage degeneration, destruction, and osteophyte formation are the main pathological features of OA [2]. Many physiological processes contribute to the development of OA, such as pyroptosis, apoptosis, and autophagy of chondrocytes. Chondrocytes undergo ferroptosis during OA, and that ferroptosis contributes to the progression of the disease [3]. Thus, inhibition of chondrocyte ferroptosis promises a potential OA therapy.

Ferroptosis is a type of iron-dependent lipid peroxidation-related cell death process that requires the accumulation of iron-dependent cellular active oxidation products [4]. Ferroptosis can lead to the progression of various diseases, including ischemia–reperfusion injury [5], neurodegenerative diseases [6], and tumors [7]. Iron is essential for cell physiological and biochemical processes, including participation in oxygen transport, DNA and ATP synthesis, and the tricarboxylic acid (TCA) cycle [8, 9]. In addition, Fe^2+^ promotes the lipid peroxidation of saturated fatty acids and the oxidative phosphorylation of mitochondria [10, 11]. The oxidative reaction induced by iron deposition leads to oxidative stress, causing protein, nucleic acid, and liposome damage directly or indirectly, which gives rise to cell damage or ferroptosis [12]. Firstly, liposome peroxidation causes damage to cell membrane composition and structure [13, 14]. In addition, liposome peroxidation produces a large amount of lethal reactive oxygen species (ROS), malondialdehyde (MDA), and peroxide dismutase (SOD), and these products can react with DNA or protein, further exerting toxic effects [15]. Glutathione peroxidase 4 (Gpx4), a lipid repair enzyme, is regulated by glutathione (GSH), which has been illustrated to block ferroptosis by reducing lipid peroxidation products and lethal reactive oxygen species accumulation [16, 17]. Activation of the antioxidant enzyme Gpx4 may have a potential to suppress ferroptosis.

Theaflavin-3,3′-digallate (TF3), a polyphenol compound extracted from black tea [18], has attracted medical attention because of its extensive pharmacological effects, such as anticancer, antiviral, antioxidant, anti-inflammatory [19], and immunomodulatory [20] effects. A previous study indicates that TF3 regulates the expression of copper transporter 1 and GSH through the wnt/*β*-catenin pathway to inhibit ovarian cancer [21, 22]. TF3 inhibited RANKL-induced osteoclast formation in bone marrow-derived macrophages (BMMs) in animal models [23]. In addition, TF3 could activate plasma kallikrein expression, reduce the deposition of fat and antagonize the oxidative damage induced by H_2_O_2_ in hepatocytes [24]. In human chondrocytes, theaflavins (TFs) reduced oxidative stress-induced apoptosis and modulated AKT activation [25]. Moreover, ferroptosis is affected by multiple signaling pathways in cells [26]. The Keap1/Nrf2 pathway is involved in glioma cell proliferation and ferroptosis [27]. MAPK/ERK/p38 [28, 29], Stat3/Nrf2/Gpx4 [30, 31], and ALK4/5 [32] signaling pathways are critical for ferroptosis induction. Despite these exciting discoveries, it remains that the role of TF3 in OA chondrocyte ferroptosis and related signaling pathway is far from elucidated. In this study, we aimed to further explore the role of TF3 in OA chondrocyte ferroptosis and elucidate its underlying mechanisms.

## 2. Materials and Methods

### 2.1. Collection of Subjects

Informed consent was received from all individual participants (including OA patients and trauma patients with femur fractures) in this study, and the sample (cartilage tissue) collection was approved by the Medical Ethical Committee of Changzhou No. 2. People's Hospital.

### 2.2. Reagents

TF3 was purchased from Shanghai YuanYe Biotech. Co., Ltd. (Shanghai, China), with an approximate purity of 98%. TF3 was dissolved in ethyl alcohol and diluted with DMEM-F12 for experiments. Collagenase II (Worthington Biochemical Corp., Lakewood, NJ, USA) was dissolved in DMEM at 2.5 mg/ml to digest the articular cartilage. Erastin, a ferroptosis activator acting on mitochondrial voltage-dependent anion channels (VADC), was purchased from Sigma–Aldrich, reconstituted in DMSO at 5 mM and stored at -20°C in the darkness. RSL3, a Gpx4 inhibitor, and deferoxamine (DFO), a ferroptosis inhibitor, were purchased from Selleckchem (Houston, TX, USA).

### 2.3. Isolation and Culture of Human Chondrocytes

Cartilage tissue specimens were obtained from the medial condyle (severely damaged joint areas) of the femur of OA patients and trauma patients with femur fracture during joint replacement surgery in the Affiliated Changzhou No. 2 People's Hospital of Nanjing Medical University. The OA severity was determined using weight-bearing anteroposterior radiographs of the affected joints according to the Kellgren and Lawrence (KL) classification (all the KL classification of all OA patients was at grade 3 in our study). These female patients with femur fractures had healthy joints. The clinical characteristics (age, gender, and disease duration) of all participants were shown in Table 1. All articular cartilage tissues were carefully minced and digested with collagenase II in serum-free Dulbecco's modified Eagle's medium (DMEM) (Gibco BRL, Grand Island, NY, USA), filtered through a 70 *μ*m cell strainer (BD, Durham, NC, USA), and extensively washed with blank DMEM/F12. Finally, chondrocytes were cultured in DMEM/F12 containing 10% fetal bovine serum (Gibco BRL, Grand Island, NY, USA), 50 *μ*g/mL ascorbic acid (AA, Sigma), 100 U of penicillin, and 100 *μ*g/ml streptomycin. When adherent cell confluence reached 90%, chondrocytes were separated. Passages 2 to 3 were used in our experiments.

### 2.4. MTS Cytotoxicity Assays

MTS (Promega, Madison, WI, USA) cytotoxicity assays were applied to assess human OA chondrocyte cytotoxicity and viability. A total of 5 × 10^3^ cells were plated in 96-well plate and allowed to attach overnight.TF3 and erastin were used to treat chondrocytes. After treatment, 10 *μ*l of MTS solution reagent was pipetted into each well of a 96-well plate. Then, the plate was incubated at 37°C for 2 hours without light, and the absorbance at 490 nm was recorded with an absorbance microplate reader (Elx808™ Bio-Tek Instruments, Winooski, VT).

### 2.5. RT–PCR Analysis

Chondrocytes were treated in different conditions, and then total RNA was extracted with the NucleoSpin RNA Kit (MN, Düren, Germany) according to the instructions. After the obtained RNA was quantified, 1 *μ*g of total RNA was taken, and reverse transcription was performed using the High Capacity cDNA Reverse Transcription kit (Applied Biosystems, Foster City, CA, USA) according to the instructions. SYBR® Select Master Mix (Applied Biosystems, Austin, TX, USA) was used to perform quantitative PCR to detect Gpx4 mRNA expression in chondrocytes, and GAPDH was selected as the internal reference. The specific primer sequences were designed by Shenggong Biotech. Co., Ltd. (Shanghai, China) and are as follows: Gpx4, 5′-GAGGCAAGACC-GAAGTAAACTAC-3′(forward) and 5′-CCGAACTGGTTACACGGGAA-3′(reverse); GAPDH, 5′-CTGGGCTACACTGAGCACC-3′(forward) and 5′- AAGTGGTCGTTGAGGGCAATG-3′(reverse). Quantitative PCR was performed using the ViiATM 7 real-time PCR system. The comparative threshold cycle method was used to determine the relative quantification of mRNA.

### 2.6. Western Blot Analysis

Cultured chondrocytes were lysed on ice with RIPA buffer (Beyotime Biotechnology, Shanghai, China) and boiled for 5 min at 99°C. A 15% polyacrylamide gel was used to separate proteins, and then proteins were transferred to a polyvinylidene fluoride (PVDF) membrane (Millipore Corp., Danvers, MA, USA). The following rabbit polyclonal antibodies were purchased from Cell Signaling Technology (Danvers, MA, USA), ABclonal Technology (Wuhan, China), and Proteintech (Wuhan, China). The information of antibodies is listed in Table S1. Rabbit polyclonal antibodies were used to detect human proteins related to the Nrf2/Gpx4 signaling pathways and ferroptosis-related proteins Slc7a11, FTH1, and HO-1. The human *β*-actin antibody was used as an internal control for protein loading, and relative expression levels were quantified using Quantity One software.

### 2.7. Propidium Iodide Stain

After different treatments, 2 *μ*l of PI solution reagent (Vazyme Biotech Co., Ltd., Nanjing, China) was pipetted into each well plate. Then, the plate was incubated at room temperature for 5 minutes without light and was observed under a fluorescence microscope (Nikon Eclipse Ti, Japan).

### 2.8. Nuclear Protein Extraction

Nuclear protein was extracted using a nuclear protein extraction kit (Beyotime, China, P0028) according to the manufacturer's protocol. The human lamin B and *β*-actin antibodies were used as the internal controls for protein loading, and relative expression levels were quantified using Quantity One software.

### 2.9. Lipid ROS Assay

Chondrocytes were incubated with 5 *μ*M C11-BODIPY^581/591^ (Thermo Fisher Scientific, USA) fluorescent probe in a serum-free medium for 30 min at 37°C in the dark and washed three times with PBS according to the modified protocol. The green and red fluorescence signals were observed under a fluorescence microscope (Nikon Eclipse Ti, Japan). The fluorescence intensity was quantified using Image Pro Plus 6.0 software.

### 2.10. Fe^2+^ Detection

The Fe^2+^ content of different groups was analyzed using a Mito-Ferrogreen Assay Kit (Dojindo, Shanghai, China) according to the manufacturer's instructions. The Fe^2+^-positive cells were green under fluorescence microscopy (Nikon Eclipse Ti, Japan), and the fluorescence intensity was quantified using Image Pro Plus 6.0 software.

### 2.11. Si-RNA Transfection Analysis

The Nuclear factor erythroid 2-related factor 2 (Nrf2) siRNA sequence was purchased from Beyotime Biotechnology (Shanghai, China) and the siRNA sequences are as follows: Si-Nrf2#1: 5′-CATTGATGTTTCTGATCTA-3′ Si-Nrf2#2: 5′-GGTTGAGACTACCATGGTT-3′; Si-Nrf2#3: 5′-GAGGCAAGATATAGATCTT-3′. Transfections were performed according to the manufacturer's instructions. The group of chondrocytes transfected with an empty vector was used as the negative control.

### 2.12. Animal Experiments

In total, 25 male SD rats, weighing 150 to 200 g, were purchased from Cavens Experimental Animal Co. Ltd. (Changzhou, China). All rats were randomly divided into five groups: control, OA, OA + Erastin, OA + Erastin+TF3, and OA + Erastin+DFO. DFO, an effective iron chelator, has been used to inhibit ferroptosis in various degenerative disease models [33]. In an OA animal model, DFO delayed the progression of primary OA. Therefore, DFO was selected as a positive control for animal experimental treatment [34]. The OA model was successfully established by medial meniscus destabilization (DMM) surgery. TF3 (1 mg/kg), erastin (1 mg/kg), or DFO (1 mg/kg) were injected into the articular cavity twice a week, and the rats were sacrificed six weeks later. The progression of OA was evaluated using the Osteoarthritis Research Society International (OARSI) scored by two blinded investigators. The effects of TF3 and DFO treatments were measured by morphological analysis and magnetic resonance imaging (MRI) examination. The animal study proposal was approved by the Animal Care Committee of Nanjing Medical University. All animal experimental procedures were performed in accordance with the Regulations for the Administration of Affairs Concerning Experimental Animals approved by the State Council of the People's Republic of China.

### 2.13. MRI Examination

Six weeks after the model operation, cartilage damage was examined by MRI.

### 2.14. Immunohistochemical Staining and Histomorphometric Measurements

Tibial and femoral tissues were separated and fixed in 10% formalin, decalcified in 10% ethylenediaminetetraacetic acid (EDTA) for 3 weeks after washing with water, and then dehydrated in graded alcohols. Specimens were embedded in paraffin and cut into 5 *μ*m serial sections. 3 sections per rat were analyzed, and all sections were from medial femoral condyles. H_2_O_2_ (3%) and BSA (5%) were used to block endogenous peroxidase activity and nonspecific binding sites, respectively. The Gpx4 primary antibodies were incubated overnight at 4°C. Next, the appropriate HRP-conjugated secondary antibody was added to the sections for incubation and counterstaining with haematoxylin at room temperature. Each glass slide was stained with HE and Masson's staining according to the manufacturer's instructions.

### 2.15. Statistical Analysis

Statistical analyses were performed using Prism8 (GraphPad Software, San Diego, CA, US). Unpaired Student's *t-*test was used for two groups; one-way ANOVA was used for more than two groups, and the Mann–Whitney *U* test was used for ranked data analysis. All quoted *p* values were 2-tailed, and those less than 0.05 were considered statistically significant.

## 3. Results

### 3.1. The Expression of Gpx4 in Cultured Chondrocytes from OA Patients

**Figure 1:**
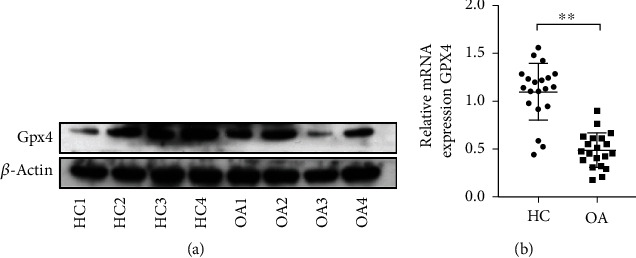
The Gpx4 expression was downregulated in cultured chondrocytes from OA patients. (a) Gpx4 protein expression in chondrocytes from trauma patients (HC1 to HC4: chondrocytes from 4 different trauma controls) and OA patients (OA1 to OA4: OA chondrocytes from 4 different OA patients). (b) The mRNA levels of Gpx4 in the chondrocytes from trauma patients (as healthy control, *n* = 20) and OA patients (*n* = 20) were examined by qPCR. Data were compared by unpaired *t*-test, ^∗∗^*p* < 0.01. HC: healthy control; OA: osteoarthritis; Gpx4: glutathione peroxidase 4.

We examined the expression of Gpx4 in cultured chondrocytes isolated from articular cartilage tissues derived from OA patients and trauma patients with femur fractures. The results showed that the mRNA and protein expression levels of Gpx4 were decreased in OA chondrocytes compared with chondrocytes from trauma patients (Figures 1(a) and 1(b)), suggesting that the ability of chondrocytes to clear ROS was largely compromised in OA.

### 3.2. TF3 Shows No Significant Cytotoxicity to Chondrocytes at Appropriate Concentrations

**Figure 2:**
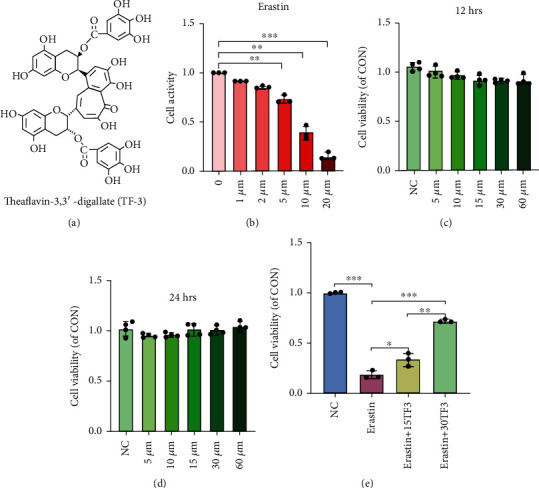
TF3 reverses erastin-induced chondrocyte viability. (a) Chemical structure diagram of TF3. (b) Chondrocytes were treated with different concentrations of erastin (1 *μ*M, 2 *μ*M, 5 *μ*M, 10 *μ*M, and 20 *μ*M) for 24 hrs. (c, d) Chondrocytes were treated with different concentrations of TF3 for 12 hrs or 24 hrs. (e) Chondrocytes were pretreated with TF3 (15 *μ*M/30 *μ*M) for 2 hours and then stimulated with erastin (5 *μ*M) for 24 hrs. The results are presented as the mean ± SD of three independent experiments, and statistical significance was determined by one-way ANOVA. ^∗^*p* < 0.05, ^∗∗^*p* < 0.01, ^∗∗∗^*p* < 0.001. NC: negative control.

The molecular structure of TF3 was shown in Figure 2(a). To evaluate the dose effect of the ferroptosis activator erastin, cultured OA chondrocytes were incubated with five different concentrations of erastin (1, 2, 5, 10, and 20 *μ*M) and the solvent DMSO as a control. As demonstrated in Figure 2(b), chondrocyte viability was decreased in a dose-dependent manner. Notably, when chondrocytes were treated with 5 *μ*M erastin, chondrocyte ferroptosis was successfully induced with a reduced viability after 12 hrs. Therefore, a 5 *μ*M concentration of erastin was used to induce ferroptosis in the subsequent experiments. Next, we used different concentrations of TF3 (5, 10, 15, 30, and 60 *μ*M) to treat OA chondrocytes for 12 hrs or 24 hrs. The MTS results showed that TF3 did not significantly affect chondrocyte viability at different concentrations (Figures 2(c) and 2(d)), suggesting that TF3 had no significant cytotoxicity to culture chondrocytes at given concentrations.

### 3.3. TF3 Reverses Erastin-Induced Cell Viability in Cultured OA Chondrocytes

To evaluate the protective role of TF3, chondrocytes were pretreated with 15 *μ*M or 30 *μ*M TF3 for 2 hrs before 24 hrs of incubation with 5 *μ*M erastin. Both concentrations of TF3 (15 and 30 *μ*M) markedly rescued erastin-induced chondrocyte viability, with a more significant effect at a higher concentration of TF3, as shown in Figure 2(e), suggesting a protective role of TF3 in erastin-induced chondrocyte.

### 3.4. TF3 Inhibits the Erastin-Induced ROS Level in OA Chondrocytes

**Figure 3:**
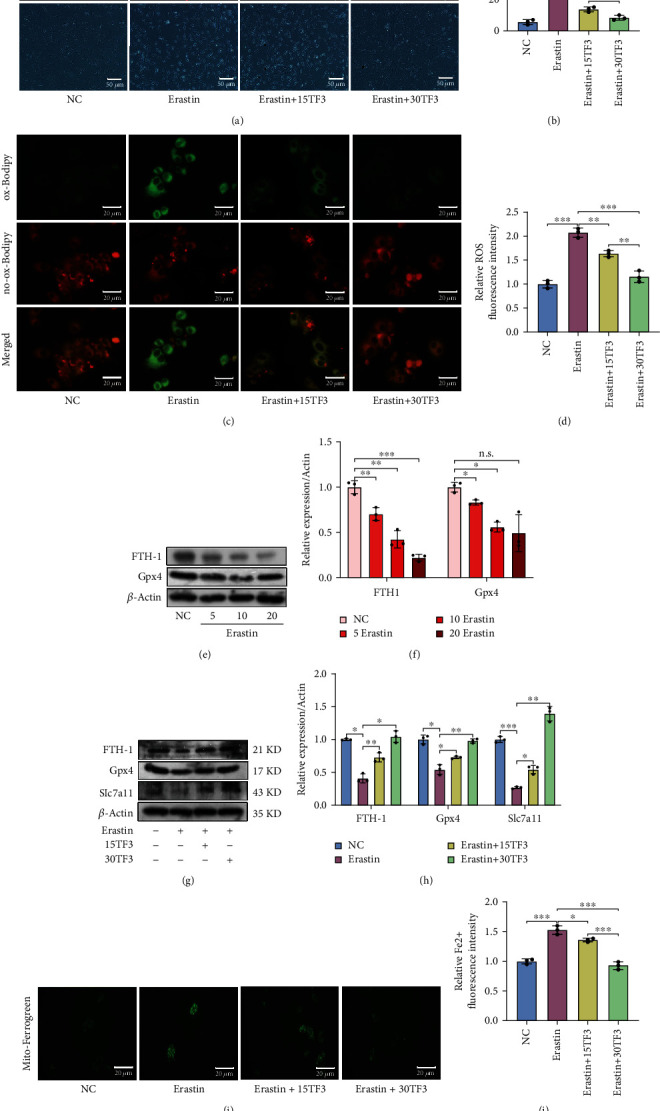
The protective effect of TF3 on erastin-induced ferroptosis in cultured OA chondrocytes. (a) Chondrocyte ferroptosis was detected by PI staining; representative images are shown. (b) Quantitative analysis of PI positive percentage. (c) Lipid ROS levels were determined by fluorescent staining; representative images are shown. (d) Quantitative analysis of lipid ROS levels in chondrocytes. (e) Levels of FTH-1 and Gpx4 in chondrocytes after stimulation by different concentrations of erastin (5 *μ*M, 10 *μ*M, 20 *μ*M) for 24 hrs were measured by western blotting. (f) Quantitative analysis for expression of FTH-1 and Gpx4 in erastin-treated chondrocytes. (g) Chondrocytes were pretreated with TF3 for 2 hours and then stimulated with erastin (5 *μ*M) for 24 hrs. The levels of FTH-1, Gpx4, and Slc7a11 were examined by western blot; representative bands are shown. (h) Quantitative analysis for the expression of FTH-1, Gpx4, and Slc7a11 in erastin-treated chondrocytes pretreated with TF3. (i) The Fe^2+^ level in mitochondria was examined by Mito-Ferrogreen staining; representative images are shown. (j) Quantitative analysis of Fe^2+^ level. The results are presented as the means ± SD of three independent experiments, and statistical significance was determined by one-way ANOVA. ^∗^*p* < 0.05, ^∗∗^*p* < 0.01, ^∗∗∗^*p* < 0.001, #*p* < 0.0001; n.s.: no significant difference. NC: chondrocytes were cultured in DMEM-F12 for 24 hrs. Erastin: chondrocytes were treated with 5 *μ*M erastin for 24 hrs. Erastin+15TF3/Erastin+30TF3: chondrocytes were pretreated with different concentrations of TF3 (15 *μ*M and 30 *μ*M) for 2 hrs and then incubated with erastin for 24 hrs. FTH1: ferritin heavy chain 1; Gpx4: glutathione peroxidase 4; Slc7a11: light chain subunit of the cystine/glutamate anticarrier.

Next, we used different concentrations of erastin to induce ferroptosis in OA chondrocytes, used PI staining to visualize the chondrocyte ferroptosis and a C11BODIPY fluorescent probe to detect intracellular ROS and lipid ROS levels, and examined ferroptosis-related protein expression by western blot. PI staining showed that chondrocyte ferroptosis was significantly promoted by erastin (Figures 3(a) and 3(b)). Both intracellular ROS and lipid ROS were accumulated in erastin-treated chondrocytes as reflected by the intensity of green fluorescence (Figures 3(c) and 3(d)). However, TF3 reduced the levels of intracellular ROS and lipid ROS and the number of PI-positive chondrocytes (Figures 3(a)–3(d)). Western blot indicated that erastin decreased ferroptosis-related protein expression, ferritin heavy chain 1(FTH-1), and Gpx4 (Figures 3(e) and 3(f)). In addition, TF3 reversed the expression of FTH-1, Gpx4, and Slc7a11 (Figure 3(g) and Figure 3(h)). Together, these observations indicated that TF3 inhibits chondrocyte ferroptosis by improving the Gpx4 expression suppressed by erastin.

### 3.5. TF3 Promotes Iron Metabolism in Cultured OA Chondrocytes

Abnormal iron metabolism is another contributor to ferroptosis, and iron deposits are found in OA [35]. Ferrogreen was used to detect the Fe^2+^ level. The results showed that Fe^2+^ was accumulated in chondrocytes treated with erastin. In contrast, TF3 reduced the accumulation as reflected by the intensity of green fluorescence (Figures 3(i) and 3(j)). In addition, Fe^2+^ was stored in FTH1, and FTH1 participated in iron metabolism progression. Western blot results showed that TF3 upregulated the expression of FTH1 in the erastin-treated chondrocytes (Figure 3(g)). Taken together, TF3 improves iron metabolism in OA chondrocytes.

### 3.6. TF3 Protects Chondrocytes from Ferroptosis via the Nrf2/Gpx4 Signaling Pathway

**Figure 4:**
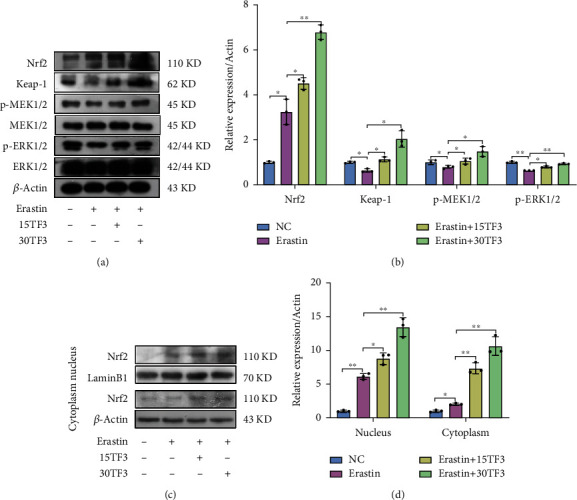
TF3 activates the Keap1/Nrf2 signaling and the MEK1/2/ERK1/2 signaling pathways in chondrocytes stimulated with erastin. (a) The levels of Nrf2, Keap1, p-MEK1/2, MEK1/2, p-ERK1/2, and ERK1/2 in chondrocytes after stimulation were examined by western blotting; representative bands are shown. (b) Quantitative analysis for the expression of Nrf2, Keap-1, p-MEK1/2, and p-ERK1/2 in chondrocytes. (c) The nucleus and cytoplasm expression levels of Nrf2 in chondrocytes were determined by western blotting; representative bands are shown. (d) Quantitative analysis for the nucleus and cytoplasm expression levels of Nrf2. The human lamin B and *β*-actin antibodies were used as the internal controls for nucleus and cytoplasm protein loading, respectively. The data are presented as the mean ± SD of three independent experiments, and statistical significance was determined by one-way ANOVA. ^∗^*p* < 0.05, ^∗∗^*p* < 0.01. NC: chondrocytes were cultured in DMEM-F12 for 24 hrs. Erastin: chondrocytes were treated with 5 *μ*M erastin for 24 hrs. Erastin+15TF3/Erastin+30TF3: chondrocytes were pretreated with different concentrations of TF3 (15 *μ*M, 30 *μ*M) and then incubated with erastin. Nrf2: nuclear factor erythroid 2-related factor 2.

**Figure 5:**
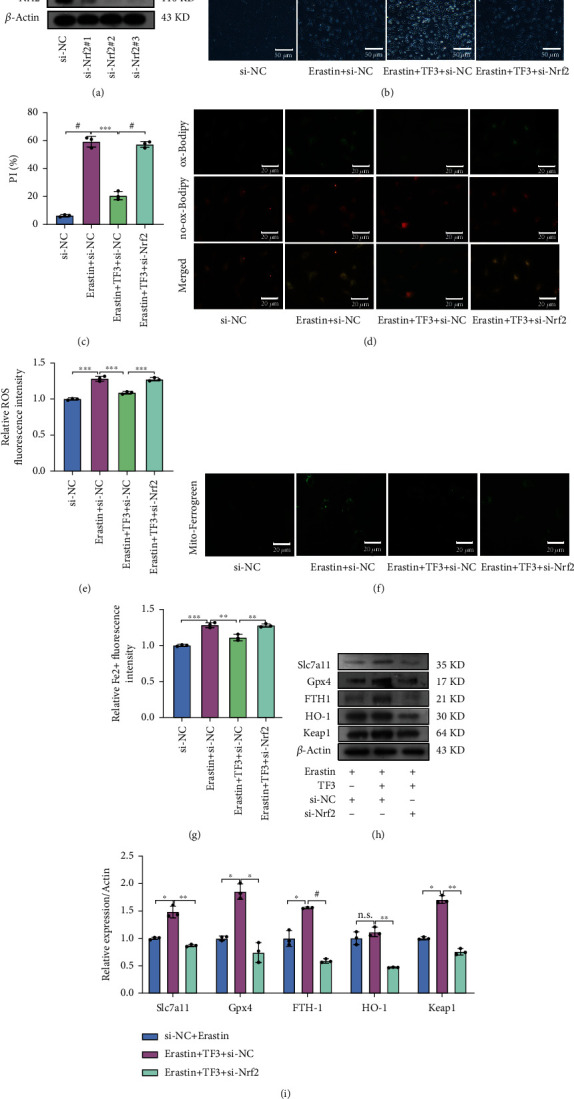
Knockdown of Nrf2 reverses TF3-antiferroptosis effects in chondrocytes stimulated with erastin. (a) The expression of Nrf2 in chondrocytes with si-RNA treatments was examined by western blotting. (b) Chondrocyte ferroptosis was detected by PI staining; representative images are shown. (c) Quantitative analysis of PI positive percentage. (d) Lipid ROS levels were evaluated by fluorescent staining; representative images are shown. (e) Quantitative analysis of lipid ROS levels in chondrocytes. (f) The Fe^2+^ levels in mitochondria were detected by Mito-Ferrogreen staining; representative images are shown. (g) Quantitative analysis of Fe^2+^ level. (h) The levels of Slc7a11, Gpx4, FTH-1, HO-1, and Keap1 in chondrocytes after stimulation were examined by western blotting; representative images are shown. (i) Quantitative analysis for the expression of Slc7a11, Gpx4, FTH-1, HO-1, and Keap1 in chondrocytes. Chondrocytes were pretreated with TF3 for 2 hours and then transfected with Nrf2 si-RNA and stimulated with erastin (5 *μ*M) for 24 hours. The data are presented as the mean ± SD of three independent experiments, and statistical significance was determined by one-way ANOVA. ^∗^*p* < 0.05, ^∗∗^*p* < 0.01, ^∗∗∗^*p* < 0.001, #*p* < 0.0001; n.s.: no significant difference.

**Figure 6:**
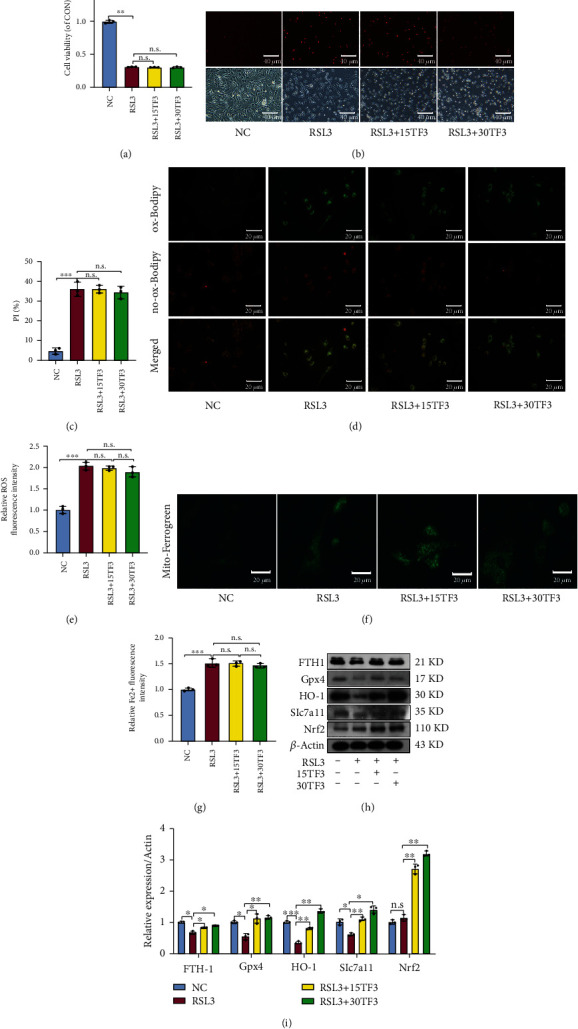
TF3 fails to reverse RSL3-induced ferroptosis in chondrocytes. (a) Chondrocyte viability was measured by MTS. (b) Chondrocyte ferroptosis was detected by PI staining; representative images are shown. (c) Quantitative analysis of PI positive percentage. (d) Lipid ROS levels were evaluated by fluorescent staining; representative images are shown. (e) Quantitative analysis of lipid ROS levels in chondrocytes. (f) The Fe^2+^ levels in mitochondria were detected by Mito-Ferrogreen staining; representative images are shown. (g) Quantitative analysis of Fe^2+^ level. (h) The levels of FTH-1, Gpx4, HO-1, Slc7a11 and Nrf2 were measured by western blot; representative bands are shown. (i) Quantitative analysis for the expression of FTH-1, Gpx4, HO-1, Slc7a11, and Nrf2 in chondrocytes. The quantitative results are presented as the means ± SD of three independent experiments, and statistical significance was determined by one-way ANOVA. ^∗^*p* < 0.05, ^∗∗^*p* < 0.01, ^∗∗∗^*p* < 0.001; n.s.: no significant difference. NC: chondrocytes were cultured in DMEM-F12 for 24 hrs. RSL3: chondrocytes were pretreated with TF3 for 2 hours and then stimulated with RSL3(10 *μ*M) for 12 hrs. FTH-1: ferritin heavy chain 1; Gpx4: glutathione peroxidase 4; HO-1: heme oxygenase-1; Slc7a11: light chain subunit of the cystine/glutamate anticarrier; Nrf2: nuclear factor erythroid 2-related factor 2.

To further investigate molecular mechanisms for TF3 in protecting chondrocytes from ferroptosis, we detected change of the signaling pathway-related proteins. TF3 significantly increased Nrf2, Keap1, p-MEK1/2, and p-Erk1/2 expressions in the total cell lysate (Figures 4(a) and 4(b)). Next, we separated the nucleus component from cytoplasm and carried out western blot analysis. A significantly increased expression of Nrf2 was observed in the nucleus and the cytoplasm upon TF3 treatment (Figures 4(c) and 4(d)). Then, we knockdown Nrf2 expression by using small interfering RNA (si-RNA) and observed the effect of TF3 on chondrocyte ferroptosis. The effectiveness of knockdown was confirmed in Figure 5(a). We observed that the combination treatment of erasin with TF3 significantly increased the PI-positive cell percentage, levels of lipid ROS, Fe^2+^ in mitochondria in the si-Nrf2 chondrocytes compared with the si-NC chondrocytes (Figures 5(b)–5(g)). On the contrary, the expression of ferroptosis-related protein (Slc7a11, Gpx4, FTH1, and HO-1) was markly decreased in erastin+TF3 + si-Nrf2 group in comparison with the erastin+TF3 + si-NC group (Figures 5(h) and 5(i)). Moreover, we further evaluated the TF3 protective role of Gpx4 by applying the Gpx4 inhibitor RSL3 to cultured OA chondrocytes for 2 hrs. The MTS results showed that TF3 failed to improve chondrocyte viability in the presence of RSL3 (10 *μ*M) (Figure 6(a)). Meanwhile, TF3 did not decrease PI-positive chondrocytes number (Figures 6(b) and 6(c)), lipid ROS (Figures 6(d) and 6(e)), and Fe^2+^ level in mitochondria (Figures 6(f) and 6(g)). However, TF3 partly reversed the expression of FTH1, Gpx4, HO-1, and Slc7a11 (Figures 6(h) and 6(i)). Collectively, these data suggested that the Nrf2/Gpx4 signaling pathway is involved in TF3-regulated chondrocyte ferroptosis.

### 3.7. TF3 Attenuates Cartilage Degradation and Increased Proteoglycans and Gpx4 Expression in a Rat OA Model

**Figure 7:**
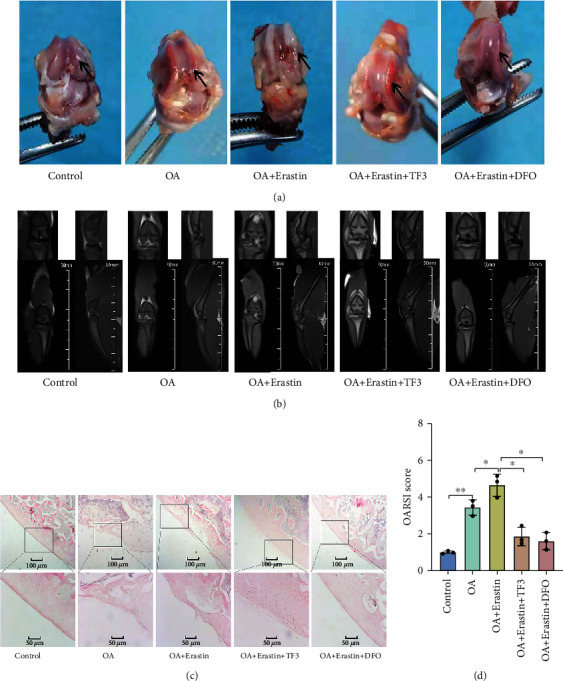
TF3 diminishes cartilage destruction in OA rat model. SD rats were divided into five groups: Control, OA, OA + Erastin, OA + Erastin+TF3, OA + Erastin+DFO. (a) The representative visual inspection of cartilage damage and representative images is shown. The arrows point to the areas of medial femoral condyles. (b) The representative MRI results of rats. (c) The representative HE staining results of different groups. (d) The progression of OA was evaluated using the OARSI scores. ^∗^*p* < 0.05, ^∗∗^*p* < 0.01. Statistical comparisons were calculated using the Mann–Whitney *U* test.

To further explore the role of TF3-regulated ferroptosis *in invo*, we established a rat OA model. The gross morphological images of the rat's knee were shown in Figure 7(a). In the OA group, the articular surface was rough and ulcerated. The degree of cartilage joint injuries was more serious in OA + erastin group, while the cartilage joint injuries were repaired in the OA + erastin+TF3 group to some extent. The MRI results illustrated that the degree of articular surface cartilage destruction in OA + erastin group was also more serious than that of in the OA group, while the degree of articular cartilage destruction was significantly alleviated in the TF3 treatment groups (Figure 7(b)). Furthermore, we also observed that cartilage damage was reversed after TF3 treatment, as showed by HE staining (Figure 7(c)) and OARSI score (Figure 7(d)). These observations suggested that articular cartilage damage might be repressed by TF3.

**Figure 8:**
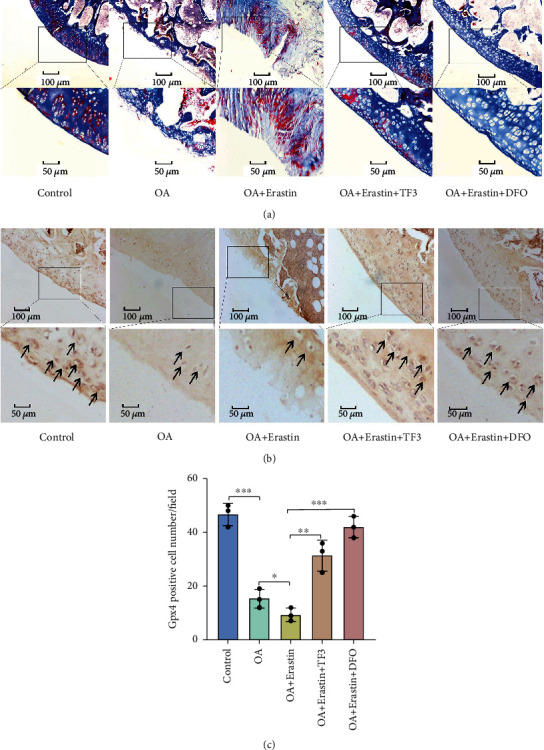
TF3 increases proteoglycans and Gpx4 expression in a rat OA model. (a) The representative Masson staining results of proteoglycans expression in different groups are shown. (b) The representative immunohistochemistry staining of Gpx4 are shown. Arrowheads indicated positive cells. (c) The number of Gpx4 positive cells per field under 100-time magnification is shown. The data are presented as the mean ± SD of three independent experiments, and statistical significance was determined by one-way ANOVA. ^∗^*p* < 0.05, ^∗∗^*p* < 0.01, ^∗∗∗^*p* < 0.001. DFO: deferoxamine.

The proteoglycans in the cartilage were gradually lost as OA progresses. We used Masson's staining to assess the changes of proteoglycans in cartilage. Intra-articular injection of 1 mg/kg TF3 significantly reversed the proteoglycans levels in the cartilage, as assessed in Figure 8(a). Immunohistochemistry staining showed that the number of Gpx4-positive chondrocytes was reduced in the OA + erastin group, but significantly increased in OA + erastin+TF3 group (Figures 8(b) and 8(c)). In summary, TF3 alleviates OA progression by Gpx4-mediated inhibition of chondrocyte ferroptosis in a rat OA model.

## 4. Discussion

In this study, we found that TF3 delayed the progression of OA and protected chondrocytes from ferroptosis via modulation of the Nrf2/Gpx4 signaling pathway. Currently, nonsteroid anti-inflammatory drugs (NSAIDS) is prescribed for treatment of OA. While it affords some protection to ferroptossis, it has noticeable side effects. Researchers have reported drug-drug interactions resulting in liver damages [36]. In erastin-induced chondrocyte ferroptosis, TF3, at nontoxic concentrations, reversed cell viability in a dose-dependent manner, suggesting that TF3 may have the fewer side effects and plays a protective role in OA chondrocyte ferroptosis.

Erastin, a ferroptosis inducer, can reduce glutathione levels by directly inhibiting cystine/glutamate antiporter system Xc - activity and activating the ferroptotic response [37], increasing ROS and iron accumulation further inducing ferroptosis [38]. Thus, we chose erastin to induce chondrocyte ferroptosis. Lipid peroxidation and iron accumulation were key factors in ferroptosis. Gpx4, an antioxidant agent, was negatively correlated with lipid peroxidation and showed protective effect on ferroptosis. Studies have indicated that the expression of Gpx4 was decreased in synovial fluid from the patients with OA and rheumatoid arthritis (RA) [39, 40], which is consistent with our findings. Its inhibition blocks intracellular iron metabolism, resulting in lipid peroxidation products and ROS accumulation and thus accelerating ferroptosis progress [41]. Additionally, ROS is related to cartilage damage in OA [42]. Consistent with previous studies, the level of Gpx4 was decreased in the erastin-treated chondrocytes. In the presence of TF3, the decreased level of Gpx4 and lethal ROS accumulation were reversed in a concentration-dependent manner, suggesting the anti-liposome peroxidation ability of TF3.

Abnormal iron metabolism contributes to ferroptosis induced by the production of ROS from the Fenton reaction [35, 43]. Divalent metal transporter 1 (DMT1, also named Slc11a2) mediates the release of Fe2^+^ from the endosome into a labile iron pool in the cytoplasm [44]. Excess iron is stored in an iron storage protein complex including ferritin light chain (FTL) and ferritin heavy chain1 (FTH1) [45]. Increased iron uptake and reduced iron storage may contribute to iron overload during ferroptosis [46]. Consistently, our results showed that the level of Fe^2+^ was increased in the erastin-treated chondrocytes and reversed by addition of TF3. In addition, TF3 promoted FTH1 expression. These observations suggested that TF3 inhibits ferroptosis by promoting iron metabolism in OA chondrocytes.

The mechanism of ferroptosis has not been fully elucidated. Cystine-glutamate antitransporter (System Xc-) [47, 48], coenzyme Q (CoQ) [49], and ASCL4-related pathways have been involved in ferroptosis [50–52]. Slc7a11, a subunit unique to system Xc, inhibits intracellular GSH depletion, iron-dependent lipid peroxidation and subsequent ferroptosis [53]. Our study showed that TF3 elevates the expression of Slc7a11, suggesting that Slc7a11 mediates TF3 anti-ferroptosis effect.

Recently, growing attention has been given to the role of the Nrf2 transcription factor in cartilage homeostasis [54]. Nrf2 is capable of regulating the basal and inducible expression of a plethora of antioxidant and detoxification enzymes, including CAT, SOD, Gpxs, heme-oxygenase1 (HO-1), NADPH, and quinone oxidoreductase1 (NQO1) [55, 56]. Pharmacological activation of Nrf2 or overexpression of Nrf2 [57] has been shown to limit IL-1*β*-induced reactive oxygen species generation and reduce the absorption of iron in chondrocytes, demonstrating the importance of Nrf2 activity on the antioxidant response in cartilage. We found that the MEK1/2, ERK1/2, and keap1/Nrf2/Gpx4 signaling pathways were activated in erastin-treated chondrocytes in the presence of TF3. Under normal conditions, the DGR region of Keap1 binds to the DLG and ETGE sequences of Nrf2, which stabilizes Nrf2 in the cytoplasm and induces Nrf2 ubiquitination and proteasome degradation [58]. Keap1 transforms its connection with Nrf2 by sensing changes in ROS, promoting nucleus Nrf2 accumulation binding with the promoter antioxidant response element (ARE) to induce downstream antioxidant proteases transcription and translation [59]. The increased ROS level was associated with the elevated Nrf2 expression, by which cells maintain oxidative stress balance [60]. These studies were consistent with the observation that after the chondrocytes were stimulated by erastin, the expressions of Nrf2 in the nucleus and lipid ROS levels in chondrocytes were all markly increased. After TF3 intervention, nucleus Nrf2 bound with the promoter ARE to induce Gpx4 expression, further scavenging lipid ROS. Further, knockdown of Nrf2 resulted in decreased expression of downstream targets (Slc7a11, Gpx4, FTH1, and HO-1), and TF3 did not completely reverse Gpx4 expression and erastin-induced chondrocyte ferroptosis in Nrf2 knockdown cells. In further support, application of the Gpx4 inhibtor RSL3 [61] caused similar results to that of Nrf2 knockdown, such as elevated lipid peroxidation, decreased cell viability, and thus increased ferroptosis. However, when chondrocytes were pretreated with RSL3, TF3 could not reverse chondrocyte ferroptosis, strongly implicating that the protective effects of TF3 on cell viability were mediated via activation of Gpx4, which protects against ferroptosis. Thus, these results suggested that the Nrf2/Gpx4-related pathway is a mediator of the protective effects of TF3 in OA chondrocytes.

Delaying articular cartilage degeneration was the key intervention method for OA treatment [62]. Systemic administration of DFO reduces cartilage lesion development in the OA model [34]. Our animal experiments results showed that TF3 could alleviate the degeneration of OA cartilage, which was manifested by a lower OARSI score and lower degree of articular cartilage destruction. Meanwhile, TF3 notably upregulated the expression of proteoglycans and antiferroptosis protein Gpx4. These *in vivo* results indicated the protective effect of TF3 on OA was similar to that of DFO.

## 5. Conclusions

In summary, we found that TF3 protects chondrocytes against erastin-induced ferroptosis via the Nrf2/Gpx4 signaling pathway activation, suggesting that TF3 might be a novel and promising therapeutic option for OA (graphical abstracts).

## Figures and Tables

**Table 1:** Characteristic of subjects investigated.

Characteristics	OA	TRUMA
Total number of subjects	20	20
Age^a^, years	65	65
25th percentile	62	61
75th percentile	72	70
Number of female/male subjects	20/0	20/0
Disease duration^a^, years	4.8	—
25th percentile	2.5	—
75th percentile	6.5	—

^a^Median.

## Data Availability

The datasets used in the present study are available from the corresponding author on reasonable request.

## Abbreviations

OA: Osteoarthritis
TCA: Tricarboxylic acid cycle
AA: Ascorbic acid
ROS: Reactive oxygen species
MDA: Malondialdehyde
SOD: Peroxide dismutase
Gpx4: Glutathione peroxidase 4
GSH: Glutathione
TF3: Theaflavin-3,3'-digallate
BMMs: Bone marrow-derived macrophages
DFO: Deferoxamine
OARSI: Osteoarthritis research society international
DMEM: Dulbecco's modified Eagle's medium
AA: Ascorbic acid
RIPA: Radioimmunoprecipitation assay buffer
PVDF: Polyvinylidene fluoride
MRI: Magnetic resonance imaging
EDTA: Ethylenediaminetetraacetic acid
DMM: Medial meniscus destabilization
Nrf2: Nuclear factor erythroid 2-related factor 2
NSAIDS: Nonsteroid anti-inflammatory drugs
DMT1: Divalent metal transporter 1
FTL: Ferritin light chain
FTH1: Ferritin heavy chain 1
System Xc–: Cystine-glutamate antitransporter
COQ: Coenzyme Q
Slc7a11: Light chain subunit of the cystine/glutamate anticarrier
NQO1: NADPH quinone oxidoreductase 1
HO-1: Heme-oxygenase 1
ARE: Antioxidant response element.

## Supplementary Materials

Supplementary Materials Table S1: the key information of all antibodies used in our study is listed, and the graphical abstracts are available in supplementary files.
