# Supplementary material for: Theaflavin-3,3′-Digallate Inhibits Erastin-Induced Chondrocytes Ferroptosis via the Nrf2/GPX4 Signaling Pathway in Osteoarthritis
Source: Oxid Med Cell Longev. 2022 Nov 17;2022:3531995. doi: 10.1155/2022/3531995 (PMC9691334; doi:10.1155/2022/3531995)
Supplement: Supplementary Materials — Table S1: the key information of all antibodies used in our study is listed, and the graphical abstracts are available in supplementary files. [file 3531995.f1.zip › Graphical abstract.docx]

Graphical abstract


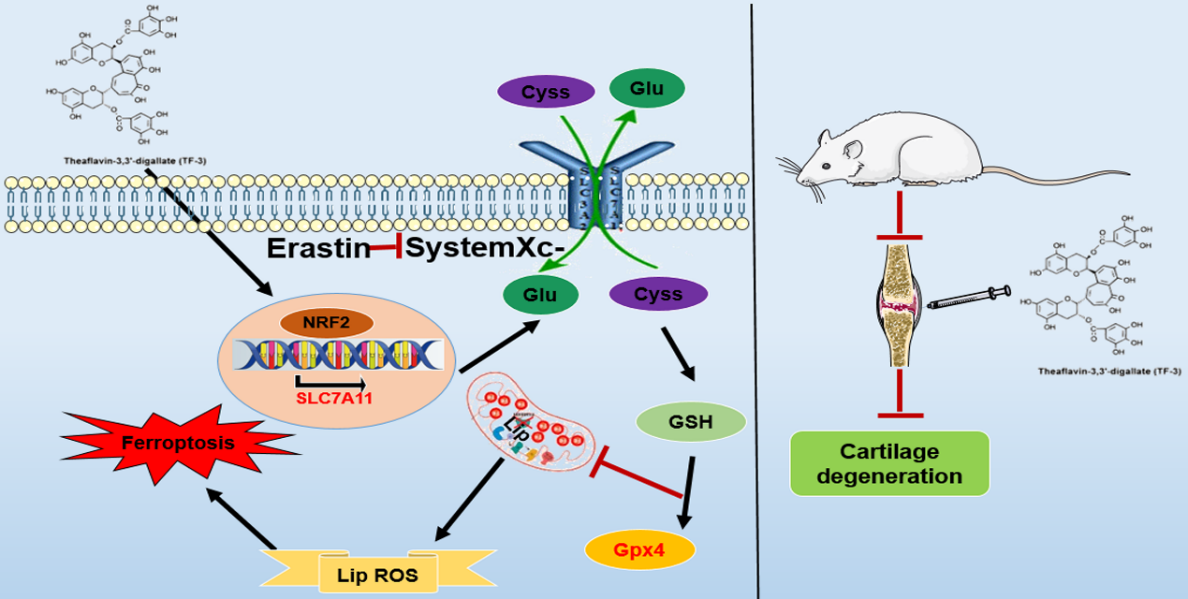


A proposed model illustrating the therapeutic effect of TF3 on osteoarthritis cartilage degeneration in rat DMM model. In vitro, Erastin blocks the system Xc- and then suppresses Gpx4, further inducing liposome peroxidation and ferroptosis. TF3-induced Nrf2 enters the nucleus and then increases the expression of Gpx4, suppressing ferroptosis. Thus, TF3 may be a potential therapeutic supplement for OA treatment.
