# Supplementary material for: Theaflavin-3,3′-Digallate Inhibits Erastin-Induced Chondrocytes Ferroptosis via the Nrf2/GPX4 Signaling Pathway in Osteoarthritis
Source: Oxid Med Cell Longev. 2022 Nov 17;2022:3531995. doi: 10.1155/2022/3531995 (PMC9691334; doi:10.1155/2022/3531995)
Supplement: Supplementary Materials — Table S1: the key information of all antibodies used in our study is listed, and the graphical abstracts are available in supplementary files. [file 3531995.f1.zip › TableS1.docx]

Table S1

The key information of all antibodies used in our study is listed in TableS1.

| antibodies | manufacturer | isotypes | catalog |
| --- | --- | --- | --- |
| Nrf2 Rabbit Polyclonal antibody | Proteintech | Rabbit IgG | 16396 |
| Gpx4 Rabbit mAb | ABclonal | Rabbit IgG | A11243 |
| Slc7a11/xCT Rabbit Polyclonal antibody | Proteintech | Rabbit IgG | 26864 |
| HO-1/HMOX1 Rabbit Polyclonal antibody | Proteintech | Rabbit IgG | 10701 |
| Keap1 Rabbit Antibody | CST | Rabbit IgG | #8047 |
| FTH1 Antibody | Abcam | Rabbit IgG | A19544 |
| ERK1/2 Rabbit mAb | CST | Rabbit IgG | #5114 |
| Phospho-p44/42MAPK(ERK1/2) Rabbit mAb | CST | Rabbit IgG | #4370 |
| p-MEK1/2 Rabbit mAb | CST | Rabbit IgG | #9154 |
| MEK1/2 Mouse mAb | CST | Mouse IgG | #4694 |
| β-Actin mouse mAb | Proteintech | Mouse IgG | 66009 |
| β-Actin Rabbit mAb | CST | Rabbit IgG | #4097 |
|  |  |  |  |
|  |  |  |  |

Lists of antibodies.
